# Pathway Editing Targets for Thiamine Biofortification in Rice Grains

**DOI:** 10.3389/fpls.2018.00975

**Published:** 2018-07-10

**Authors:** Anu P. Minhas, Rakesh Tuli, Sanjeev Puri

**Affiliations:** Department of Biotechnology, University Institute of Engineering and Technology, Panjab University, Chandigarh, India

**Keywords:** biofortification, CRISPR, gene editing, pathway, rice, thiamine, vitamin B1

## Abstract

Thiamine deficiency is common in populations consuming polished rice as a major source of carbohydrates. Thiamine is required to synthesize thiamine pyrophosphate (TPP), an essential cofactor of enzymes of central metabolism. Its biosynthesis pathway has been partially elucidated and the effect of overexpression of a few genes such as *thi1* and *thiC*, on thiamine accumulation in rice has been reported. Based on current knowledge, this review focuses on the potential of gene editing in metabolic engineering of thiamine biosynthesis pathway to improve thiamine in rice grains. Candidate genes, suitable for modification of the structural part to evolve more efficient versions of enzymes in the pathway, are discussed. For example, adjacent cysteine residues may be introduced in the catalytic domain of *thi4* to improve the turn over activity of thiamine thiazole synthase 2. Motif specific editing to modify promoter regulatory regions of genes is discussed to modulate gene expression. Editing *cis* acting regulatory elements in promoter region can shift the expression of transporters and thiamine binding proteins to endosperm. This can enhance dietary availability of thiamine from rice grains. Differential transcriptomics on rice varieties with contrasting grain thiamine and functional genomic studies will identify more strategic targets for editing in future. Developing functionally enhanced foods by biofortification is a sustainable approach to make diets wholesome.

## Introduction

Over 2 billion people suffer from different types of malnutrition worldwide (Global Nutrition Report, 2016). The role of vitamins in stress tolerance, growth and development has been reported in human and plants (Hellmann and Mooney, 2010; Galluzzi et al., 2013; Colinas and Fitzpatrick, 2015). Except vitamins B3 (niacin) and D, human body is incapable of synthesizing other vitamins. Because of high water solubility and heat sensitivity, majority of B-type vitamins and vitamin C are lost during cooking, resulting into their deficiency in diets. The average storage span of vitamin B1 in human body is only about 18 days and therefore, it needs to be replenished from diet regularly (Wooley, 2008). This review focuses on the enrichment of food grains with vitamin B1 (also called thiamine or aneurin), taking rice as the model crop and lays emphasis on pathway engineering through genome editing.

## Mini-review

Consuming less than recommended daily dose of vitamin B1 (0.2 to 1.5 mg in infants to adults) results in beriberi/Wernicke-Korsakoff syndrome (Joint FAO/WHO Expert Consultation, 2004) (Supplementary Table 1). Women develop symptoms of edema and paresthesia whereas infants show acute cardiac failure, gastrointestinal symptoms and lactic acidosis, resulting in increased mortality (Keating et al., 2014; Moulin et al., 2014; Porter et al., 2014; Barennes et al., 2015). Limited information is available on prevalence of thiamine deficiency worldwide. Among underdeveloped countries, in South East Asia alone, 27–78% mothers and 15–58% children are reported deficient in thiamine (Keating et al., 2014; Whitfield et al., 2017). Consuming high dose of thiamine is reported to reduce the development of nuclear cataract and lens opacification. In Type 2 diabetic patients, thiamine therapy is recommended to reverse the process of micro albuminuria (Cumming et al., 2000; Jacques et al., 2005; Rabbani et al., 2009). Fruits, nuts, fermented food products, majority pulses, seafood, meat and meat products are good sources of thiamine. Contrary to this, majority staple crops including rice are either deficient in thiamine or store it in inedible plant parts (USDA, 2013). Information about thiamine content and estimated fold increase required in different food types to meet recommended daily allowance (RDA) of thiamine is given in Supplementary Table 2.

## Rationale behind thiamine deficiency in developing countries

Approximately 375 million people worldwide and around 30% of Indian population is vegetarian (Chemnitz and Becheva, 2014). Rice (*Oryza sativa*) alone contributes to 27–80% of food energy in developing countries (FAO, 2011). Thiamine content in rice (per 100 g of grains) ranges from 0.053 mg (Poland variety, polished) to 3.03 mg (Indonesian variety, unpolished) (Supplementary Table 3). Elimination of rice aleurone layer (where thiamine is predominantly stored) reduces thiamine content to 0.11/100 g in polished rice grains which further reduces to 0.01g/100 g in cooked rice (Sautter et al., 2006; Mohd Fairulnizal et al., 2015). Consequently, exclusive feeding on white rice diet plan results in thiamine deficiency in human.

## Approaches to thiamine enhancement in rice

In developing countries, chemical fortification of food grains with thiamine encounters problems of lack of food processing industries, affordability, scalability, stability during transport, storage and public distribution (Sautter et al., 2006; Zhu et al., 2007; Mayer et al., 2008). Mononitrate and hydrochloride derivatives of thiamine used to fortify rice grains are heat sensitive and result in taste alteration (Steiger et al., 2014). Biofortification is the most easily applicable and sustainable seed technology in even poorly managed agriculture. Biofortification approaches can be designed to enhance total thiamine content and its bioavailability through thiamine re-localization in crop plants (WHO/NHD, 1999). However, such strategies require detailed knowledge of involved metabolic pathway, transportation and localization of thiamine in edible plant parts.

## Thiamine biosynthesis pathway in rice

**Figure 1:**
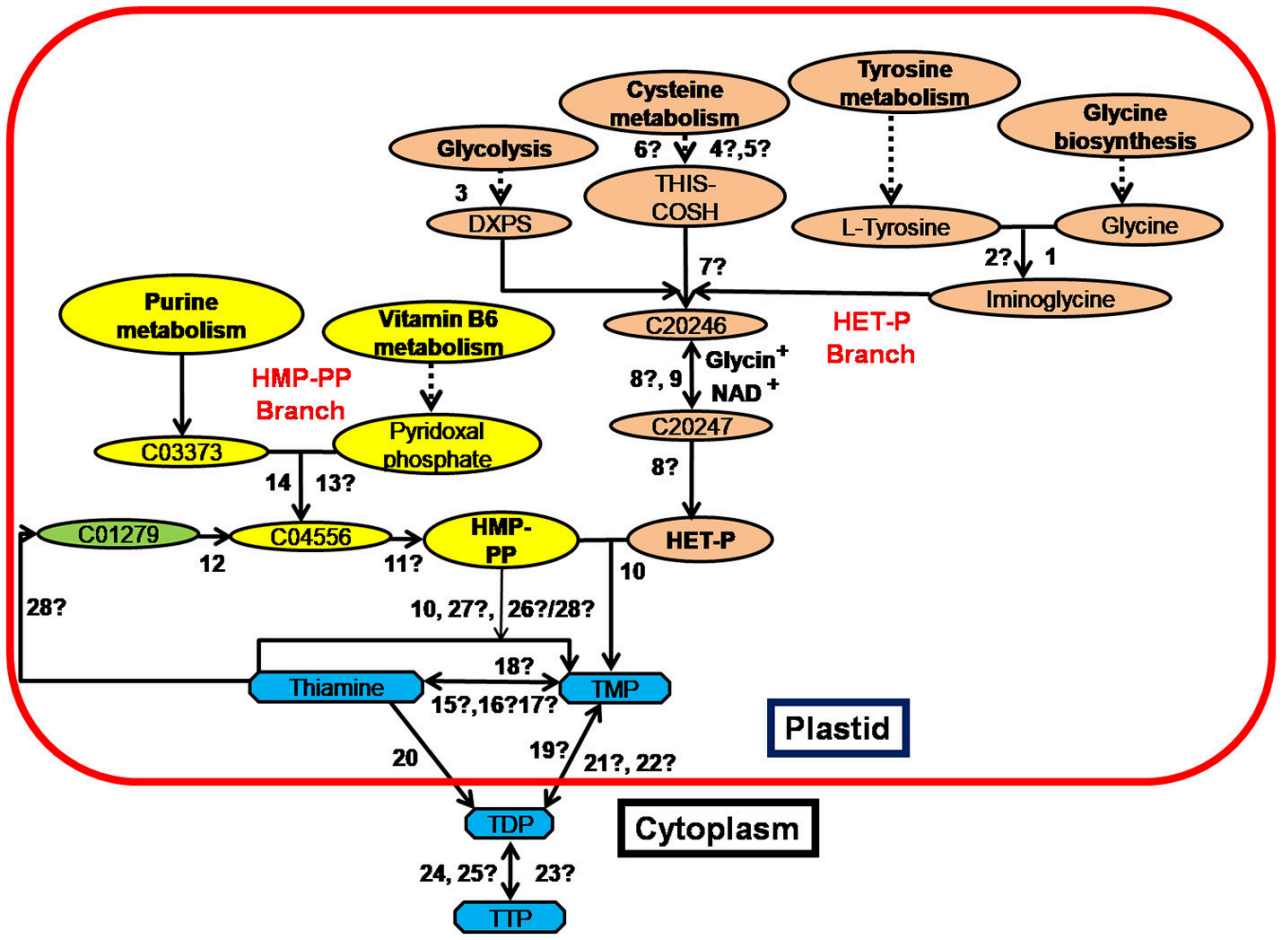
Summary of the Thiamine biosynthesis pathway in *E. coli, A. thaliana*, and *O. sativa* (modified from Kyoto encyclopedia of genes and genomes database). The two major branches HMP-PP (2-methyl-4-amino-5-hydroxymethylpyrimidine pyrophosphate) and HET-P (4-methyl-5-(β-hydroxyethyl) thiazole phosphate) are highlighted in different colors. The intermediates of the pathway are shown in the figure. Details of the enzymes numbered from 1 to 28 are provided in Supplementary Table 4. Unidentified enzymes in rice genome have been indicated with a question mark (?). Discontinuous lines indicate that all steps in the pathway are not shown in the figure.C20246, (2-[(2R,5Z)-2-Carboxy-4-methylthiazol-5(2H)-ylidene] ethyl phosphate); C20246, (2-[(2R,5Z)-2-Carboxy-4-methylthiazol-5(2H)-ylidene] ethyl phosphate); C03373, (Aminoimidazole ribotide); C01279, (4-Amino-5 hydroxymethyl-2-methylpyrimidine); C04556, (4-Amino-5-hydroxymethyl-2-methylpyrimidine phosphate); DXPS, 1-Deoxy-D-xylulose 5-phosphate; TMP, Thiamine monophosphate; TDP, Thiamine diphosphate; TTP, Thiamine triphosphate.

Thiamine in plants exists as phosphate derivatives, like thiamine monophosphate (TMP), thiamine diphosphate (TDP) and thiamine triphosphate (TTP). TDP, also called as thiamine pyrophosphate (TPP) is the functional form while un-phosphorylated thiamine is the transportable form of thiamine in plants and human (Bettendorff et al., 2007; Gangolf et al., 2010). Major steps of thiamine biosynthesis pathway have been described in different organisms (Belanger et al., 1995; Machado et al., 1996; Chabregas et al., 2001; Wang et al., 2006; Ajjawi et al., 2007; Raschke et al., 2007). Based on the information available in KEGG (Kyoto encyclopedia of genes and genomes) database, the thiamine biosynthesis pathway in *Escherichia coli, Arabidopsis thaliana* and *O. sativa* is summarized in Figure 1 and enzymes involved are listed in Supplementary Table 4. The key enzymes of HET-P (4-methyl-5-(β-hydroxyethyl) thiazole phosphate) branch of thiamine biosynthesis pathway are thiazole synthase (encoded *by thiG*), thiazole tautomerase (encoded *by tenI)* and thiamine thiazole synthase 2 *(thi4* family gene*)*. Thiazole synthase and thiazole tautomerase are reported in *E. coli* while thiamine thiazole synthase 2 has only been reported in *Arabidopsis* and *O. sativa*. Phosphooxymethyl pyrimidine kinase, pyrimidine precursor biosynthesis enzyme and phosphomethyl pyrimidine synthase encoded by *thiD, thi5* and *thiC* respectively are involved in HMP-PP (2-methyl-4-amino-5-hydroxymethylpyrimidine pyrophosphate) branch of the pathway. The *thiD* and *thi5* are present exclusively in *E. coli* whereas *thiC* has been identified in all three organisms. HET-P and HMP-PP condensation to TMP in chloroplast is catalyzed by the ubiquitous thiamine biosynthetic bifunctional enzyme (encoded by *th1*) (Figure 1 and Supplementary Table 4). Synthesized TMP is transported across chloroplast membrane in the form of free thiamine and is phosphorylated to TPP by cytosolic *tpk1* encoding thiamine pyrophosphokinase (Ajjawi et al., 2007; Pourcel et al., 2013). The *adk1*-encoded kinase phosphorylates TPP to TTP in the cytosol. Phosphatases are responsible for dephosphorylating TPP to TMP and further to thiamine. One such phosphatase encoded by *th2* has been identified in *Arabidopsis* (Mitsuda et al., 1979; Molin and Fites, 1980; Rapala-Kozik et al., 2009). The unidentified enzymes in rice genome are highlighted with a question mark (?) in Figure 1 and Supplementary Table 4.

## Genome editing with CRISPR-Cas9 endonuclease system

A number of approaches for editing DNA sequences have become available in recent years (Pabo et al., 2001; Boch et al., 2009; Moscou and Bogdanove, 2009). However, those requiring protein designing (such as zinc finger nucleases, ZFN and transcription activator like effector nuclease, TALEN) are less versatile, more expensive and complex to apply than the RNA designing based approach. The RNA based approach utilizes clustered regularly interspaced short palindromic repeats (CRISPR) and Cas9 nuclease for gene editing. Though the sequence target to be edited can be modified by any of these approaches, the level of precision, efficiency and operative details are different for each method. In the CRISPR-Cas9 system, a guide RNA is designed against the target sequence in gene (Gaj et al., 2013; Ji et al., 2015; Jones, 2015; Pan et al., 2016). gRNA-Cas9 complex scans the target DNA for protospacer sequence by searching a protospacer adjacent motif (PAM) and triggers double stranded break in the target sequence (Bolotin et al., 2005; Marraffini and Sontheimer, 2008; Mojica et al., 2005; Garneau et al., 2010; Cong et al., 2013; Hsu et al., 2014). Rapid progress has been made in using CRISPR-Cas9 system in plants for modification of traits (Supplementary Table 5). Two CRISPR-Cas9 edited plants (white button mushroom resistant to browning and *Camelina sativa* with enhanced omega-3 oil content) have been de-regulated in the US market (Waltz, 2016, 2018). One concern associated with CRISPR/Cas9 editing is the off targeting of very similar sequences and homeologs in polyploid crops (Wang et al., 2014; Li et al., 2015, 2016a; Jiang et al., 2016; Andersson et al., 2017). To improve specificity, strategies such as selecting for high homology between the guide sequence and target region near the PAM site, backcrossing mutant plant to wild parents and promoter selection for expressing gRNA and Cas9 have been suggested (Wang et al., 2015; Chen et al., 2016; Li et al., 2016b; Murugan et al., 2017). Another concern associated with CRISPR/Cas9 is the need for high throughput screening of the mutant lines. A PCR based high-resolution fragment analysis method (HRFA) with 1 bp resolution limit has been developed to facilitate screening of multiple lines (Li et al., 2016b).

## Attempts for genetic modification of thiamine biosynthesis pathway

Among limited attempts made toward thiamine enrichment, none has led to sufficient increase in thiamine content (Supplementary Table 6). The overexpression of native and riboswitch-truncated versions of *thiC* in *Arabidopsis* has been reported to show 1.5 and 3.0 fold increase respectively in total seed thiamine. Riboswitch is a conserved sequence at 3′untranslated region of pre-mRNA in *thiC* and *thi1*, which binds TPP (at 500 mM binding constant in *Arabidopsis*) and induces conformational change resulting in splicing of intron 2. The intron splicing eliminates polyadenylation signal, making the transcript unstable and therefore negatively regulates thiamine biosynthesis (Croft et al., 2007). The *thiC* null mutant exhibits severely compromised growth phenotype with low thiamine, TMP and TPP content (Kong et al., 2008; Beatty et al., 2009; Bocobza et al., 2013). Feeding studies with pathway precursor/s increase free thiamine and TPP content in *thiC* null mutant but the increase in thiamine content is almost equivalent to natural thiamine content in wild type (Pourcel et al., 2013). These findings suggest *thiC* as an important regulatory enzyme of thiamine biosynthesis pathway. However, *thiC* overexpression alone is not enough to achieve sufficient thiamine enrichment in plants. Simultaneous overexpression of *thi1/thi4* (*osdr8* in rice) and *thiC* in *Arabidopsis* increases total thiamine content in leaf and seed by 3.4 and 2.6 fold respectively. Resultant plants exhibit stress tolerance phenotype similar to the wild type. Overexpressing *thi4* and *thiC* in rice increases grain thiamine content by ~5 fold but display no altered resistance to *Xanthomonas oryzae* pv. *oryzae* (Pourcel et al., 2013; Dong et al., 2015, 2016).

## Target genes for thiamine biofortification through gene editing

The above studies suggest that the enrichment of precursors of HET-P and HMP-PP branches in plastid is a pre-requisite to grain thiamine enhancement. To achieve HET-P enrichment, enzymes with a question mark (?) from number 1–8 in Figure 1 (information in Supplementary Table 4) need be identified and characterized in rice. Among these, *thi1/thi4* enzyme in rice and *S. cerevisae* catalyzes synthesis of thiazole moiety by transferring sulfur from its conserved cysteine residue (cys205) to thiazole precursor. In this reaction, catalytic cysteine residue transforms to a dehydroalanine (Dha) residue by losing its sulfur. Restoration of dehydroalanine (Dha) residue back to cysteine has not been reported, rendering *thi4* inactive in single turnover reaction and therefore, limits HET-P biosynthesis (Chatterjee et al., 2011; Pourcel et al., 2013). Park and Raines (2001) have reported the formation of vicinal disulfide turn by oxidation of adjacent cysteine residues in a protein. The adjacent residues act as an artificial “redox switch” to modulate the conformational stability and catalytic activity of the protein (Carugo et al., 2003). Adjacent disulfide bonds have naturally been reported in many proteins (Ghosh et al., 1995; Gehrmann et al., 1998; Wang et al., 2000). The analysis of sequences of *thi4* variants in different rice varieties will identify the target sites for disulphide engineering to improve *thi4* turnover by facilitating electron exchange. For HMP-PP enrichment, *thiC*, the key enzyme of the branch has to be overexpressed, followed by *th1* overexpression to accelerate TMP condensation. Synthesized TMP has to be transported efficiently across chloroplastic membrane to cytosol and to seed endosperm by overexpressing *tpk* encoding thiamine pyrophosphokinase. In seed, genes coding for phosphatases have to be activated to accumulate thiamine in unphosphorylated (bioavailable) form.

Due to divergent expression of HMP-PP and HET-P branches in different plant tissues, seed storage of thiamine is determined by the source to sink movement of thiamine and/or its precursor molecules via membrane transporters. High level of expression of *thi4* with negligible *thiC* expression is reported in maize endosperm and is a direct indicator of pyrimidine import from adjoining tissues (Robbins and Bartley, 1937; Bonner and Buchman, 1938; Rodionov et al., 2002). Martinis et al. (2016) reported *put3* in *A. thaliana*, encoding transporter involved in transporting thiamine and polyamine across phloem tissue. The *put3* mutant shows impaired thiamine distribution among tissues, affecting plant growth. Thiamine is stored as stable complex with thiamine binding proteins (TBP) in peripheral layers of grains. Such proteins have been reported in many crops (maize, oat, faba bean, and garden pea) (Gołda et al., 2004; Pourcel et al., 2013; Blancquaert et al., 2015). However, removal of peripheral layers from cereal grains during milling process results in the loss of vitamin binding proteins and thus, the bound vitamin (Gołda et al., 2004; Pourcel et al., 2013). Therefore, emphasis must be on the identification of tissue specific thiamine transporters required for efficient translocation of thiamine or precursors from photosynthetic tissue to seed and then to the endosperm along with thiamine binding proteins for stable localization.

A number of *cis* elements required for endosperm specific expression have been identified in promoter sequences (Wu et al., 1998; Kawakatsu et al., 2008; Le et al., 2008; Nie et al., 2013). The expression of *thi1* and *thiC* is also influenced by abiotic factors such as salt, flood and light (Ribeiro et al., 2005). However, the current knowledge of epigenetic response elements in promoter sequences is insufficient to design epigenetic regulation as an approach to maximize gene expression in endosperm for enhanced thiamine bioavailability. Thus, the strategies for biofortification will improve, with increase in knowledge of thiamine biosynthesis pathway genes, their regulatory mechanisms, interacting proteins, thiamine specific kinases, phosphatases, tissue specific transporters, and vitamin stabilizing proteins in rice.

## Need to identify gene-editing targets by analyzing rice varieties with contrasting thiamine content

Goyer and Sweek (2011) have reported significant differences in transcriptome data of two potato cultivars differing two folds in their thiamine content. Differential expression of genes in thiamine contrasting varieties to identify editing targets is an important area that has not received sufficient attention. Only scanty information is available on thiamine associated diversity in rice genotypes (Supplementary Table 3) but no transcriptome information is available for these varieties. Therefore, extensive screening of diverse rice genotypes, including landraces for grain thiamine content can give more clues about genes with differential expression, helping in modifying thiamine biosynthesis pathway in rice. Identification of genes encoding differentially expressed transporters by comparative tissue transcriptomics approach (assisted by laser capture microscopy) in contrasting rice varieties is one such example.

## Expression analysis of genes of thiamine biosynthesis pathway for editing by CRISPR-Cas9 system

Tissue specific transcriptome data provides transcriptional pattern of genes in different tissues. Among available rice expression databases such as “OryzaExpress,” “TENOR” (transcriptome encyclopedia of rice), “PLEXdb” (plant expression database) and “RiceXPro” etc., we notice that only “RiceXPro” provides expression values in FPKM (fragments per kilobase of transcript per million mapped reads) for different tissues and growth stages of Nipponbare rice (Hamada et al., 2011; Sato et al., 2011a, 2013; Dash et al., 2012; Takehisa et al., 2012; Kawahara et al., 2016). Hence, the “RiceXPro” database was utilized in this analysis. Even “RiceXPro” does not provide expression information on all the genes of thiamine biosynthesis pathway. The analysis showed level of expression of *ncs1* gene encoding putative allantoin permease (cell membrane transporter), *thiC* gene encoding phosphomethylpyrimidine synthase, a *thiC* family gene encoding pentatricopeptide and three *tpk* genes encoding thiamine pyrophosphokinase paralogs in different tissues. All above genes except *tpk2* (2.18487 FPKM in endosperm) show negligible expression in endosperm and whole seed (Table 1).

**Table 1:** Analyses of the tissue specific expression of genes of thiamine biosynthesis pathway derived from “RiceXPro” database (Sato et al., 2011a,b, 2013).

**Gene Name**	**Locus ID**	**Chrom. number**	**Chrom. locus**	**No. of amino acids encoded**	**Corresponding orthologs present**	**Maximum expression (tissue/ FPKM)**	**Expression level in endosperm/whole seed (FPKM)**	**Expression level in other tissues (FPKM)**
*tpk1*	LOC_Os01g70580	1	40871884–40868708	265	*Brachypodium* and *Sorghum*	Pistil /10.2538	0/1.00868	Post-emergence inflorescence (5.71105)
*tpk2*	LOC_Os01g25440	1	14398598–14391999	268	*Brachypodium, Sorghum* and maize	Anther/29.5105	2.18487/3.53966	Embryo at 25DAP (8.76654)
*tpk3*	LOC_Os05g30454	5	17649664–17644844	268	*Brachypodium, Sorghum* and maize	Shoot/12.8129	0/0	Pre-emergence inflorescence (2.57715)
Putative *ncs1*	LOC_Os02g44680	2	27077280–27079020	539	*Brachypodium, Sorghum* and maize	Pre-emergence inflorescence /2.86182	0/0	Pistil (1.86176)
*thiC*	LOC_Os03g47610	3	26952048–26959200	640	*Brachypodium, Sorghum* and maize	Post-emergence inflorescence /139.85	0.304733/1.40095	20 day old leaves (93.1279), Shoots (41.0685)
*thiC* family protein	LOC_Os12g17080	12	9777751–9782552	539	*Brachypodium, Sorghum* and maize	Post-emergence inflorescence/6.85087	0.219805/1.504	Embryo 25 DAP (5.35801)

The promoter sequence regions of three rice *tpk* variants were analyzed for the presence of endosperm-specific *cis* elements. The sequences showed the presence of AACA motif, ACGT motif, Prolamin box and TATA box in *tpk3* promoter sequence, in the region 300 bp upstream of the transcription start site (Thompson et al., 1997; Higo et al., 1999; Supplementary Table 7 and Supplementary Figure 1). An approach to use CRISPR-Cas9 for editing is illustrated by taking the example of *tpk3* gene, with the objective of achieving its expression in endosperm tissue. The Supplementary Figure 1, shows the 300 bp promoter region of *tpk3* allele (LOC_Os05g30454). Though other motifs are present, the critical motif GCN4 is missing. This can strategically be introduced upstream of the above motifs by editing at a selected position. A 20 bp gRNA sequence with NGG as the PAM is shown, as a suitable target site for gRNA (Supplementary Figure 1). The target site has been selected for its absence of off-site targets in rice genome using E-CRISP and Cas-OFFinder tools respectively (Bae et al., 2014; Heigwer et al., 2014). Three to four nucleotides upstream of the PAM, the DNA is cleaved by Cas9. By transient or stable transformation with the gRNA and Cas9, a GCN4 box can be introduced at the point of editing by co-transformation with oligonucleotides designed to promote homologous recombination in the target region. The absence of GCN4 motif is in agreement with negligible expression of *tpk3* in rice endosperm. The resultant edited gene carrying the GCN4 with other endosperm specific motifs will be expected to show endosperm specific expression of *tpk3* gene.

In conclusion, complete information about genetic components of thiamine biosynthesis pathway and their regulatory mechanisms is required to redesign the pathway for high level expression of thiamine in grain endosperm. Thiamine biosynthesis pathway mentioned for rice in this study identifies a list of uncharacterized genes (*thiH, thiG/thiS, thiF, thiD, phoA, aphA, thiK etc*.). Genes coding for thiamine specific transporters, kinases, and phosphatases need to be identified in rice genome. These are required to carry thiamine precursors and synthesize thiamine to endosperm in bioavailable form. Identification of thiamine stabilizing proteins and their level of expression in rice endosperm are important to ensure high level of stable thiamine in agronomically elite varieties. Genes have been shortlisted for modifying structural part to encode more efficient version of proteins (*thi1/thi4*) and the expression level of the candidate gene can be modulated by promoter engineering (*tpk, thiC* and *thi4/th1*). To transport synthesized thiamine or its precursors efficiently to endosperm of rice grain, introducing *cis* elements to the promoters by promoter engineering has been suggested for transporters such as Tpk, Put3 or Ncs1. Tissue specific transcriptome information needs to be generated for rice genotypes with contrasting levels of thiamine, to identify novel target genes encoding rate limiting enzymes of thiamine biosynthesis pathway. The CRISPR-Cas9 technology has made gene editing much simpler than ever before, and therefore is highlighted as the method of choice.

## Author contributions

AM initiated the project, collected and analyzed data, wrote the manuscript, RT reshaped the title, designed technical details, provided expert feedback, reviewed the article critically, SP commented and reviewed the article.

### Conflict of interest statement

The authors declare that the research was conducted in the absence of any commercial or financial relationships that could be construed as a potential conflict of interest.
