# Corrigendum: Pathway Editing Targets for Thiamine Biofortification in Rice Grains

**DOI:** 10.3389/fpls.2018.01813

**Published:** 2019-01-21

**Authors:** Anu P. Minhas, Rakesh Tuli, Sanjeev Puri

**Affiliations:** Department of Biotechnology, University Institute of Engineering and Technology, Panjab University, Chandigarh, India

**Keywords:** biofortification, CRISPR, gene editing, pathway, rice, thiamine, vitamin B1

In the original article “(Dong et al., 2016)” was cited in the supplementary data but not in the published version of the original article. On Dr. Aymeric Goyer note, the citation has now been inserted in the section “Attempts for genetic modification of thiamine biosynthesis pathway” and should read: “Overexpressing *thi4* and *thiC* in rice increases grain thiamine content by ~5 fold but display no altered resistance to *Xanthomonas oryzae* pv. *oryzae* (Pourcel et al., 2013; Dong et al., 2015, 2016)”.

The authors apologize for this error and state that this does not change the scientific conclusions of the article in any way. The original article has been updated.

## Conflict of Interest Statement

The authors declare that the research was conducted in the absence of any commercial or financial relationships that could be construed as a potential conflict of interest.
