# Supplementary material for: Pathway Editing Targets for Thiamine Biofortification in Rice Grains
Source: Front Plant Sci. 2018 Jul 10;9:975. doi: 10.3389/fpls.2018.00975 (PMC6048418; doi:10.3389/fpls.2018.00975)
Supplement: Supplementary file 1 [file Data_Sheet_1.doc]

Supplementary Material

Pathway Editing Targets for Thiamine Biofortification in Rice Grains

Anu Priya Minhas^*^, Rakesh Tuli, Sanjeev Puri

Department of Biotechnology, University Institute of Engineering and Technology (UIET), Panjab University (PU), Chandigarh, India

***Correspondence:**Anu Priya Minhas, Department of Biotechnology, University Institute of Engineering and Technology (UIET), Panjab University (PU), Sector-25, Chandigarh, India, 160014.

*e-mail*: annuminhas@gmail.com, priyacoa@rediffmail.com

**Supplementary Table 1. Recommended daily intake of vitamin B1 (thiamine or aneurin) in infants to adults (WHO/FAO Report, 2004)**

| **Group** | **Thiamine RDA (mg/day)** |
| --- | --- |
| Infants and children | 0.2 |
| 0–6 months | 0.3 |
| 7–12 months | 0.5 |
| 1–3 years | 0.6 |
| 4–6 years | 0.9 |
| Adolescents | 1.2 |
| 10–18 years | 1.2 |
| Adults F/M | 1.4 |
| Pregnant women and Lactating women | 1.5 |

**Supplementary Table 2. Thiamine content (mg/100g) in different food types of animal and plant origin (Paul and Southgate, 1979; Thomas, S. 1970). Details of serving size of each meal type (g/meal), amount of each food type required to fulfill RDA and fold increase in thiamine content required for each food type are given.**

|  | **Thiamine content (mg/100g)** | **Serving size (g/meal)** | **Gram of food needed to meet thiamine RDA (0.2-1.5)** | **Fold increase in food required to achieve in food, RDA** |
| --- | --- | --- | --- | --- |
| Wheat, starch | 0.2 | 11 | 110-800g | 10-80 |
| Wheat chapati | 0.3 | 60 | 65-550 g | 9 |
| Rice polished | 0.07 | 160 | 300-2100g | 2-13 |
| Rice, boiled | 0.01 | 160 | 1600-12800g | 13-100 |
| Eggs | 0.7 | 100 | 30-230g | No Need |
| Fish and sea foods | 1.3 | 100 | 15-110g | No Need |
| Fruits | 0.6 | 100 | 170-1300g | No Need |
| Meat and meat products | 0.35 | 100 | 60-480g | No Need |
| Milk and milk products | 0.3 | 200 | 65-530g | No Need |
| Vegetables | 0.015 | 50 | 1330-10650g | 8-64 |

**Supplementary Table 3. Natural thiamine content reported in rice varieties (mg/100g of rice grains)**

| **Rice varieties** | **Thiamine content (mg/100g)** | **Reference** |
| --- | --- | --- |
| Pusa Basmati-1 | 0.070±0.001 | Sood et al., 2006 |
| Haryana Basmati-1 | 0.073±0.001 |
| Basmati 370 | 0.072±0.001 |
| PR-106 | 0.064±0.002 |
| Tarori Basmati | 0.068±0.001 |
| HKR 91-406 | 0.066±0.001 |
| HKR 92-401 | 0.065±0.00l |
| HKR 93-401 | 0.065±0.002 |
| HKR 93-402 | 0.065±0.001 |
| HKR 91-455 | 0.064±0.001 |
| HKR 92-455 | 0.063±0.002 |
| HKR 92-447 | 0.064±0.003 |
| Njavara (brown rice) | 0.52 ± 0.01 | Deepa et al., 2008 |
| Jyothi (brown rice) | 0.35 ± 0.01 |
| IR 64 (brown rice) | 0.40 ± 0.08 |
| Poland variety | 0.053 | Lebiedinska and Szefer, 2006 |
| Karnataka rice cultivar | 0.2-0.45 | Rimal Isaac et al., 2012 |
| Kerala rice cultivar | 0.4-0.5 |
| Tamil Nadu rice cultivar (Kullakar) | 0.5 |
| B102674PN623222332 Indonesian variety | 3.03 | Indrasari et al., 2014 |
| B10876HMR2 Indonesian variety | 2.13 |
| B10531EKN12PN142 Indonesian variety | 1.33 |
| B12411ERS*­121 Indonesian variety | 1.21 |

**Supplementary Table 4. Details of the enzymes of thiamine biosynthesis pathway as depicted in Figure 1. The homologs present in *E. coli*, *A. thaliana* and *O. sativa* are shown*.* The unidentified enzymes in *O. sativa* are designated with a question mark (?).**

| **No. assigned in Figure 1** | **Protein encode** | **EC of enzyme** | **Gene name** | | |
| --- | --- | --- | --- | --- | --- |
|  |  |  | ***E. coli*** | ***A. thaliana*** | ***O. sativa*** |
| 1 | **Glycine oxidase** | 1.4.3.19 | *thi****O/****thiG* | ND | LOC4345597 |
| 2 | 2-iminoacetate synthase | 4.1.99.19 | *thiH* | ND | ? |
| 3 | 1-deoxy-D-xylulose-5-phosphate synthase | 2.2.1.7 | ND | ***dxpsI*** | ***Dxps1*, *claI*** |
| 4 | Sulfur carrier protein ThiS adenylyltransferase | 2.7.7.73 | *thiF* | ND | ? |
| 5 | Cysteine desulfurase | 2.8.1.7 | ND | *****nfs2* (chloroplastic)/**** *nfs1/csd/iscS* | ? |
| 6 | tRNA uracil 4-sulfurtransferase | [**2.8.1.4**](http://www.genome.jp/dbget-bin/www_bget?ec:2.8.1.4) | ND | *thiI* | ? |
| 7 | Thiazole synthase | 2.8.1.10 | *thiG* | ND | ? |
| 8 | **Thiazole tautomerase** | 5.3.99.10 | *tenI* | ND | ? |
| 9 | Thiamine thiazole synthase 2 | [2.4.2.60](http://www.genome.jp/dbget-bin/www_bget?ec:2.4.2.60) | ND | thi4 family | *thi4*, *thi1* |
| 10 | **Probable thiamine biosynthetic bifunctional enzyme (Chloroplastic)** | 2.5.1.3 | *thiE* | *th1* | LOC_Os12g09000 |
| 11 | Phosphooxymethylpyrimidine kinase (Hydroxy methyl pyrimidine phosphokinase) | 2.7.4.7 | ***thiD*** | ND | ? |
| 12 | **Probable thiamine biosynthetic bifunctional enzyme (chloroplastic)** | 2.7.1.49 | ***thiD*** | *th1* | LOC_Os12g09000 |
| 13 | Pyrimidine precursor biosynthesis enzyme | K18278 | *thi5* | ND | ? |
| 14 | Phosphomethyl pyrimidine synthase (chloroplastic) | 4.1.99.17 | *thiC* | *thiC* | LOC_Os03g47610  putative *thiC* |
| 15 | **Alkaline phosphatase** | 3.1.3.1 | *phoA* | ND | ? |
| 16 | **Acid phosphatase** | 3.1.3.2 | *aphA* | *pap17* | ? |
| 17 | **Thiamine phosphate phosphatase**  **Bifunctional Th2 protein (mitochondrial)** | 3.1.3.100 | ND | th2 | ? |
| 18 | **Thiamine kinase** | 2.7.1.89 | *thiK* | ND | ? |
| 19 | Thiamine-monophosphate kinase | 2.7.4.16 | *thiL* | ND | ? |
| 20 | Thiamine pyrophosphokinase | 2.7.6.2 | ND | *tpk1* | *tpk1* -LOC_Os01g70580*tpk2*-LOC_Os01g25440*tpk3*-LOC_Os05g30454 |
| 21 | Nucleoside-triphosphate phosphatase | 3.6.1.15 | ND | ND | ? |
| 22 | Hydrolases | 3.6.1.- | ND | ND | ? |
| 23 | Thiamine triphosphatase | 3.6.1.28 | ND | ND | ? |
| 24 | **Adenylate kinase 1 (chloroplastic)** | 2.7.4.3 | *adk* | *adk1* | LOC_Os03g03820 |
| 25 | Thiamine diphosphate kinase | 2.7.4.15 | ND | ND | ? |
| 26 | Thiamine pyridinylase (pyrimidine transferase/ thiaminase I) | 2.5.1.2 | ND | ND | ? |
| 27 | Hydroxyethylthiazole kinase | 2.7.1.50 | *thi****M*** | *thi****M*** | ? |
| 28 | Bifunctional Th2 protein (mitochondrial) | 3.5.99.2 | ND | *th2* | ? |

ND-Not Determined

**Supplementary Table 5. Recent cases** **of CRISPR-Cas9 applications to plants for trait modification**

| **Source of gene** | **Name of target Gene** | **Gene function** | **Cas9 promotor** | **sgRNA promotor** | **Transformation method** | **Mutational efficiency (%)** | **Final outcome of gene editing** | **Reference** |
| --- | --- | --- | --- | --- | --- | --- | --- | --- |
| 1. *A. thaliana* and *Nicothiana benthamiana* | *gn1a, dep1, gs3, ipa1* | Regulators of grain number, panicle architecture | Maize ubiquitin promotor | INA | *Agrobacterium* transformation in embryogenic calli | 42.5 | Enhanced grain number, dense eract panicle and large grain size respectively | Li et al. (2016a) |
| *Nicotiana tabacum* cv. BY2 cells | *xylT* and *fucT* | β (1,2)-xylosyltranferase and the α(1,3)-fucosyltransferase | CaMV35S | AtU6 | *Agrobacterium* transformation | 30 | Exhibited N-linked glycans lacking β (1,2)-xylose and/or α (1,3)-fucose | Hanania et al. (2017) |
| *Triticum aestivum* | *Tamlo* homolog | Repress resistance pathway to powdery mildew | ZmUbi | TaU6 | Particle bombardment of immature embryos | 5.6 | Heritable resistance to powdery mildew | Wang et al. (2014) |
| *Glycine max* | *als1* | Encode acetolactate synthase involved in amino acid biosynthesis pathway | EF1A2 | Soyabean U6-9-1 | Particle bombardment | 59-76 | Chlorsulfuran resistant soyabean | Li et al. (2015) |
| *O. sativa* | *Oserf922* | ERF transcription factor | Ubi | OsU6a | *Agrobacterium* mediated electroporation | 42 | Enhancing blast resistance | Wang et al. (2016) |
|  | *sbeI* and  *sbeIIb* | Starch branching enzyme | OsU3 | OsU3 | *Agrobacterium* mediated electroporation | 26.7 to 40% | High amylose rice | Sun et al. (2017) |
| *O. sativa* (Japonica rice) | *csa* | Carbon starved anther | INA | INA | *Agrobacterium* mediated | 50 | Male sterile phenotype | Li et al. (2016b) |
| *Camelina sativa* | *fad2* | Key enzyme involved in synthesis of polyunsaturated fatty acids | CaMV35SP | U9P | *Agrobacterium* floral dip | 60 | Significant increase in oleic acid content | Jiang et al. (2016) |
|  | *Csdgat1*- and *Cspdat1* | Involved in triacylglycerol (TAG) synthesis pathway in developing seeds | CaMV35SP | U6-26P | *Agrobacterium* mediated electroporation | INA | Seed with reduced oil content and altered fatty acid composition | Aznar-Moreno and Durrett (2017) |
| *A. thaliana* | *elf4E* and *ilf (iso)4E* | Target site of ethyl methanesulfonate (EMS) | PcUbi4-2 | At U6-26 | *Agrobacterium* mediated electroporation | 70.9 | Resistant to Turnip mosaic virus (TuMV) | Pyott et al. (2016) |
| *S. tuberosum* | *Stiaa2* | INA | CaMV35S | StU6 | *Agrobacterium* mediated | INA | INA | Wang et al. (2015) |
|  | *als1* | Acetolactate synthetase | U6 Pol II | U6 Pol II | Geminivirus meditated transformation of leaves | 60 | Reduced herbicide susceptibility phenotype | Butler et al. (2016) |
|  | *gbss* | Granule bound starch synthase | CaMV35S | U6 | Protoplast transfection | 67 | Altered amylase synthesis | Anderson et al. (2017) |
| *Phaselous vulgaris* | *Bean yellow dwarf virus* (BeYDV) genome | INA | INA | INA | INA | INA | Reduced virus copy number | Baltes et al. (2015) |
| *Hordeum vulgare* | *eng* | Endo-*N*-acetyl-β-D-glucosaminidase gene | Ubi | U6Os | *Agrobacterium* mediated and particle bombardment | 78 | Modification of N-glycans | Kapusi et al. (2017) |
| *Salvia miltiorrhiza* | *Smcps1* | Diterpene synthase gene involved in tanshinone biosynthesis | CaMV35S | AtU6 | A. rhizogenes mediated transformation | 42.3 | Tanshinones reduction | Li et al. (2017) |
| *Papaver somniferum L.* cv. Ofis-95 | 4'OMT2 | Regulates the biosythesis of benzylisoquinoline alkaloids | CaMV35S | AtU6 | Agrobacterium mediated leaf infiltration | 80 | Decrease of S-reticuline levels | Alagoz et al. (2016) |

INA- Information not available

**Supplementary Table 6. Summary of genetic engineering approaches reported for thiamine biofortification in rice and *A. thaliana***

| **Gene** | **Type of manipulation** | **Promoter used** | **Targeted Plant** | **Observed effects** | **Remark (s)** | **Reference** |
| --- | --- | --- | --- | --- | --- | --- |
| OsDR8 (*thi1* homolog) | Repression | INA | Rice | Decreased thiamine content | Confirmed  *thi1* as essential target enzyme for thiamine accumulation | Wang et al. (2006) |
| *thiC* | Overexpression | Ubiquitin1 or CaMV35S | *A. thaliana* | ~1.5 fold increase in thiamine level | - | Kong et al. (2008) |
| *alaAT* | Overexpression | Root specific | Rice | 2.17 fold increase in *thiC* expression | Increased biomass of resultant transgenic plants | Beatty et al. (2009) |
| *thiC gene with mutated* riboswitch (A515G in the 3-UTR) | Overexpression | Native /ubiquitin1 | *A. thaliana* | ~1.5-3 fold in TMP in *Arabidopsis* leaves and seeds | Less photosynthetic activity, showed stunted growth with delayed flowering | Bocobza et al. (2013) |
| *A. thaliana thi1* and *thiC* | Overexpression | Constitutive promoter | *A. thaliana* | 3.4- and 2.6-fold total thiamine in leaf and seeds respectively | Tolerance to biotic and abiotic stress equal to wild type | Dong et al. (2015) |
| *thi1* and *thiC* | Overexpression | INA | Rice | ~5 fold increase in thiamine content in leaves and unpolished grains | No improvement to biotic stress tolerance like *A. thaliana*. | Dong et al. (2016) |
| *A. thaliana put3* | Deletion | INA | *A. thaliana* | Altered thiamine distribution among tissues | Defective plant growth and development | Martinis et al. (2016) |

**Supplementary Table 7. Analysis of 0.3kb promoter region sequence (upstream to transcription start site, TSS) in the three *tpk* alleles in rice. The postions of the endosperm-specific motifs, GCN4, Prolamin box (P box), AACA motif, ACGT motif, TATA Box, Y patch and CATGCA (Wu et al., 1998; Le et al*.*, 2008; Kawakatsu et al., 2008; Nie et al., 2013) are shown. The numerals indicate the position of the motif, from the transcription start site.**

| ***tpk* variants**  ***cis* elements** | **GCN4,**  TGA(G/C)TCA | **Prolamin box,**  (AAAG) **(Number of motifs; position)** | **AACA motif (Number of motifs; position)** | **ACGT motif (Number of motifs; position)** | **TATA Box**  (TATTTAA variants, **position**) | **Y Patch,**  (TTCTCTC and TCCTC) | **CATGCA box** |
| --- | --- | --- | --- | --- | --- | --- | --- |
| LOC_Os01g70580 (*tpk1*) | Ab | 1; -130 | Ab | 1; -42 | TATAAG (-229),  TATTTA (-162),  CATAAAT (-93),  TATTTA (-17) | Ab | Ab |
| LOC_Os01g25440 (*tpk2*) | Ab | 2; -104, -110 | Ab | Ab | Ab | Ab | Ab |
| LOC_Os05g30454 (*tpk3*) | Ab | 1; -276 | 4; -253, -240, -12, +6 | 1; -161 | TATAAG (-205) | Ab | Ab |


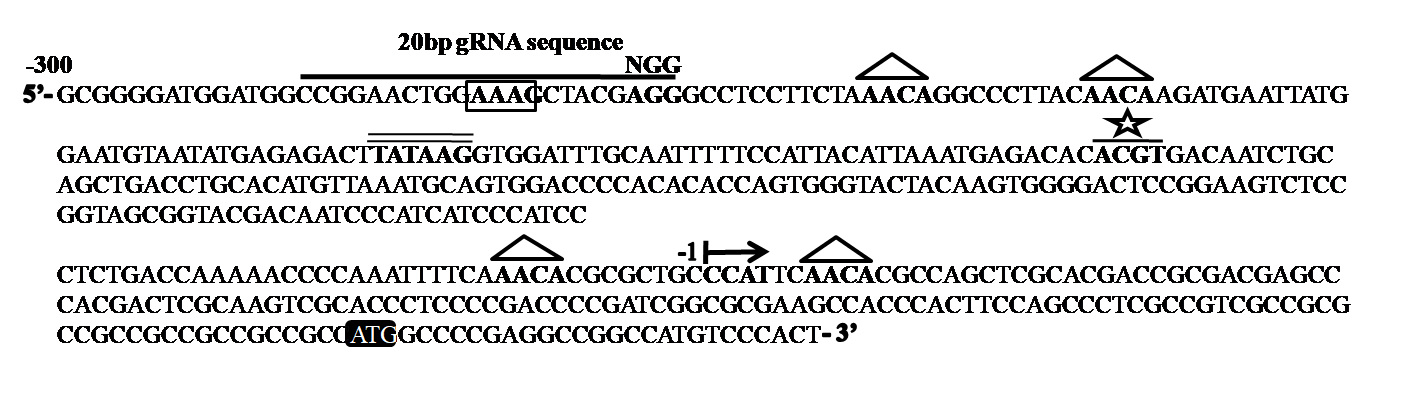


**Supplementary Figure 1. Sequence of 0.3kb promoter region, upstream of the *tpk3* allele (LOC_Os05g30454) in rice, illustrated to propose how CRISR can be deployed to edit the promoter region for the expression of *tpk*3 at high level in endosperm tissue of rice grain. The numerals indicate the nucleotide position from transcription start site (TSS) of the *tpk3* allele. The TSS is highlighted in bold with an arrow, and the translation initiation site start (ATG) is highligthed in black. The 20bp gRNA sequence with NGG as the PAM sequence is shown with single bar on top. This sequence is the putative target site for gRNA. Three to four nucleotides upstream of PAM, the DNA is cleaved by the Cas9. A desired motif, like GCN4 can be inserted at such a site by oligonucleotide directed homologous recombination. The location of other endosperm expression specific notifs in the promoter region proposed for editing is shown. The Prolamin box (AAAG sequence) is shown in bold, encased in a rectangular box, AACA motif is shown in bold with an upright triangle and ACGT motif is bold with single bar and a star. The Y Patch and CATGCA box** are **absent in all the three *tpk* promoter sequences analyzed.**

**SUPPLEMENTARY REFERENCES**

Baltes, N. J., Hummel, A. W., Konecna, E., Cegan, R., Bruns, A. N., Bisaro, D. M., et al. (2015). Conferring resistance to geminiviruses with the CRISPR–Cas prokaryotic immune system. *Nat. Plants* 2015:15145. doi: 10.1038/NPLANTS.2015.145

Butler, N. M., Baltes, N. J., Voytas, D. F., and Douches, D. S. (2016). Geminivirus mediated genome editing in potato (*Solanum tuberosum* L.) using sequence specific nucleases. *Front. Plant Sci*. 7:1045. doi: 10.3389/fpls.2016.01045

Deepa, G., Singh, V., and Naidu, K. A. (2008). Nutrient composition and physicochemical properties of Indian medicinal rice-Njavara. *Food Chem*. 106, 165–171. doi: 10.1016/j.foodchem.2007.05.062

Dong, W., Thomas, N., Ronald, P. C., and Goyer, A. (2016). Overexpression of thiamin biosynthesis genes in rice increases leaf and unpolished grain thiamin content but not resistance to *Xanthomonas* *oryzae pv. Oryzae. Front. Plant Sci.* 7, 1–11. doi: 10.3389/fpls.2016.00616

Indrasari, S. D., Ardhiyanti, S. D., and Abdullah, B. (2014). Study of milling process and its effect on vitamin B1 and folic acid contents on lowland rice promising lines*. Indo. J. Agric. Sci*. 15, 2. doi: 10.21082/ijas.v15n2.2014.p79-85

Lebiedinska, A., and Szefer, P. (2006). Vitamin B in grain and cereal grain food, soy products and seeds. *Food Chem*. 95, 116–122. doi: 10.1016/j.foodchem.2004.12.024

Paul, A. A., and Southgate, D. A. T. (1979). *The Composition of Foods*. Amsterdam; New York, NY; Oxford: Elsevier/North-Holland Biomedical Press.

Pyott, D. E., Sheehan, E., and Molnar, A. (2016). Engineering of CRISPR/Cas9-mediated potyvirus resistance in transgene-free *Arabidopsis* plants. *Mol. Plant Path*. 17, 1276–1288. doi: 10.1111/mpp.12417

Rimal Isaac, A. R., Nair, A. S., Varghese, E., and Chavali, M. (2012). Phytochemical, antioxidant and nutrient analysis of medicinal rice (*Oryza sativa* L.) varieties found in South India. *Adv. Sci. Lett*. 5, 1–5. doi: 10.1166/asl.2012.2174

Sood, D. R., Deka, S. C., and Singh, A. P. (2006). Nutritional quality of basmati rice genotypes. *J. Dair. Foods* H5. 25, 1–7. Government Publishing Service. 5th Edn.

Thomas, S. (1970). *Tables of composition of Australian foods*. Canberra, Australian Government Publishing Service. 5th Edn.

Wang, F., Wang, C., Liu, P., Lei, C., Hao, W., Gao, Y., et al. (2016). Enhanced rice blast resistance by CRISPR/Cas9-targeted mutagenesis of

the ERF transcription factor gene OsERF922. *PLoS ONE* 11:e0154027. doi: 10.1371/journal.pone.0154027
